# Predicting receptor-ligand pairs through kernel learning

**DOI:** 10.1186/1471-2105-12-336

**Published:** 2011-08-11

**Authors:** Ernesto Iacucci, Fabian Ojeda, Bart De Moor, Yves Moreau

**Affiliations:** 1 SCD-ESAT, Department of Electrical Engineering, Katholieke Universiteit Leuven, Kasteelpark Arenberg 10, Leuven, 3001, Belgium

## Abstract

**Background:**

Regulation of cellular events is, often, initiated via extracellular signaling. Extracellular signaling occurs when a circulating ligand interacts with one or more membrane-bound receptors. Identification of receptor-ligand pairs is thus an important and specific form of PPI prediction.

**Results:**

Given a set of disparate data sources (expression data, domain content, and phylogenetic profile) we seek to predict new receptor-ligand pairs. We create a combined kernel classifier and assess its performance with respect to the Database of Ligand-Receptor Partners (DLRP) 'golden standard' as well as the method proposed by Gertz *et al. *Among our findings, we discover that our predictions for the tgfβ family accurately reconstruct over 76% of the supported edges (0.76 recall and 0.67 precision) of the receptor-ligand bipartite graph defined by the DLRP "golden standard". In addition, for the tgfβ family, the combined kernel classifier is able to relatively improve upon the Gertz *et al. *work by a factor of approximately 1.5 when considering that our method has an *F*-measure of 0.71 while that of Gertz *et al. *has a value of 0.48.

**Conclusions:**

The prediction of receptor-ligand pairings is a difficult and complex task. We have demonstrated that using kernel learning on multiple data sources provides a stronger alternative to the existing method in solving this task.

## Background

Regulation of cellular events is initiated, often, via extracellular signaling. Extracellular signaling occurs when a circulating ligand interacts with one or more membrane-bound receptors. Identification of receptor-ligand pairs is thus an important and specific form of protein-protein interaction (PPI) prediction. While the problem of predicting PPI has been highly studied, little effort has been placed on the sub-problem of predicting receptor-ligand interactions.

A tremendous amount of research has been applied to the problem of predicting PPI. Foremost in the field has been prediction via phylogenetic profile analysis. Generally, this type of investigation studies the similarity of the phylogenetic history of a protein *A *and its putative protein partner *B*. Broadly speaking, the assortment of these types of phylogenetic studies examine the most accurate measure of similarity between the phylogenetic histories of *A *and *B*. Findings from these studies support the idea that proteins which interact have similar phylogenetic profiles, as these proteins should co-adapt as they are under the same evolutionary pressures [1,2].

Bhardwaj *et al. *[3] make use of the phylogenetic information strategy while introducing expression data to predict PPI. Their findings support the idea that integrating gene expression profile and phylogenetic information increases the accuracy of predictions than phylogenetic analysis alone. The rational of using co-expression as an indicator of PPI originates from the observation that proteins which interact for the purpose of performing a similar function are likely to be co-expressed as they will need to be present at the same time to carry out their common biological activity [4,5].

The notion of combining expression and phylogenetic information to predict PPI is clearly a step in a direction which leads us to consider a wider variety of data integration. Here we propose a framework in which other sources of data (such as domain content) can be applied to a kernel solution to the problem. The rational behind incorporation of domain content information is as follows: as certain domains are known to interact, it is self evident that this data would provide additional insight into the problem of determining receptor-ligand pairs.

One of the groups which have tackled the receptor-ligand prediction task is Gertz *et al. *[6]. In their work, they match members of the chemokines and tgfβ ligand families with their respective receptor families. They used distance matrices of the receptors and ligands families to measure similarity between the groups. Through a Metropolis Monte Carlo optimization algorithm, they explored and scored possible matches between the two matrices, until they reached optimal solutions. While their work was successful, they rely only on phylogenetic distance matrices, here we propose the integration of multiple data sources to help make more accurate matches.

We look into the use of creating a combined kernel classifier to carry out this learning task. While many kernel-based machine learning techniques have been applied to the PPI task [7,8], it has hitherto never, to our knowledge, been used on the receptor-ligand problem. Kernel learning provides the means to utilize enigmatically related data (such as expression measures, domain content, etc.) and perform classification in higher dimensional space via kernel methods. In our work, we apply the least-squares support vector machines (LS-SVM) method based on the conclusions by Suykens *et al. *[9] which shows this implementation to be robust. As different data sources are used, separate LS-SVM kernel classifiers were built and the combined output used to provide a final result.

While the task addressed here is the predictions of successful protein ligand-receptor pairings, a related area of research is the protein-chemical interaction prediction task for which kernels have, sometimes, been applied. For example, Nagamine *et al. *[10] approach this task through the use of a SVM trained on vector representations of protein-chemical pairs. Building on this, Jacob *et al. *[11] demonstrate the utility of using hierarchical kernels to match proteins with chemical ligands in a similar learning task. This line of research was then further advanced by Bleakley *et al. *[12] who introduce the use *of bipartite local models *which use kernels to successfully predict several reported drug-target interactions.

We first describe our combined kernel classifier method to predict receptor-ligand pairings. We then present the bipartite-graph we derive from our findings and compare it to our "Golden Standard" and to results previously published by Gertz *et al. *[6]. Following this, we interpret the performance of our method with respect to this comparison. To conclude, we discuss the benefits and limitations of our method and possible future directions for this work.

## Methods

### 1. Problem Formulation

Our objective is to predict candidate receptor-ligand pairs; more specifically, we seek to create a method to identify known pairs as well as to determine putative pairs for further research. Our method involves using multiple data sources (expression, phylogenetic, and protein-domain content information), computing separate kernels for each data type, creating LS-SVM classifiers and combining the results to predict receptor-ligand pairs.

### 2. Data Sources

For the datasets used, our setting is as follows, candidate receptor and ligand sequences were retrieved for seven species (*Rattus norvegicus, Mus musculus, Homo sapiens, Pan troglodytes, Canis familiaris, Cavia porcellus*, and *Bos taurus*) from ensemble build 51 [13]. The sequences were then aligned using ClustalW [14]. Once aligned, the sequences were edited so as to eliminate the positions which had the 5% lowest substitution scores across the seven orthologous sequences. The pair-wise alignment score was then taken for each possible species to species comparison between the edited orthologous sequences (as seven species are used, a total of 21 pair-wise comparisons for each candidate is created). The distance scores form a phylogenetic vector [2] which will then be used to create the phylogenetic kernel.

The expression for the candidates was taken from the well-known GNF human expression atlas (79 tissues) [15], the data was normalized (values were mean-zeroed and the standard deviation was set to one) and was further transformed into the expression kernel.

The domain content of each candidate protein (receptor or ligand) was taken from the Interpro Database [16]. A vector for each candidate protein was created where the presence of a protein domain was indicated with a'1' and the absence of a domain was indicated by a '0'. This data was then transformed to create the domain content kernel.

### 3. Kernels and LS-SVM Classifier

The above mentioned data matrices (phylogenetic, expression, and domain content) were used to create three kernels for each receptor-ligand family. LS-SVMs [9] were trained using the three kernels to predict outcomes for receptor-ligand pairs known from our Database of Ligand-Receptor Partners (DLRP) "Golden Standard".

**Figure 1:**
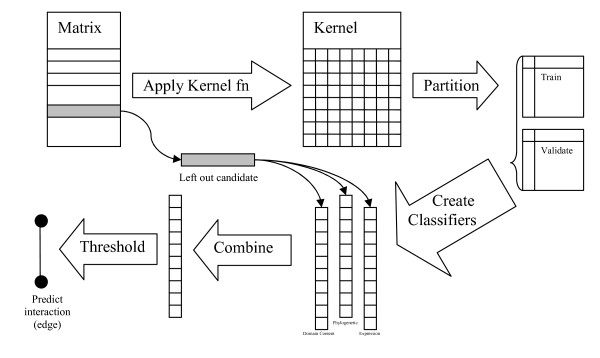
**Work flow of the combined kernel classifier**. For each candidate, Data was partitioned into training and validation sets and parameters were tuned using a 5 fold validation strategy. The final output of the classifiers was achieved by a leave one out strategy. The classifier values were combined for a final result and a threshold was applied to determine which values are predicted edges in the receptor-ligand bipartite graph.

Our kernel function measures the similarity between two proteins *A *and *B *(K(*A,B*)), one a candidate receptor (*A*) and the other a candidate ligand (*B*). Our LS-SVM classifier is a binary predictor which assigns new examples in "interacting" or "non-interacting" classes. Creating the kernels from these matrices involved trials with different kernel functions (radial based function, linear, and polynomial), linear functions being found to give the best performance in all cases. A combined kernel approach was also considered but empirical results determined that a combined classifier approach was preferable. The regularization parameters for the LS-SVMs were tuned using a two tier grid search which, at first, uniformly ranged from 10^-6 ^to 10^6 ^in 10^1 ^unit steps followed by a second finer search with 10^0.1 ^unit steps. For each candidate, data was partitioned into training and validation sets and parameters were tuned using a 5-fold validation strategy (300 random partitions of the data were performed). The final output of the classifiers was achieved by a leave-one-out strategy. The classifier values were scaled (minimum set to zero, maximum set to one) and combined, as defined in (1), for a final result. Figure 1 provides an overview of the workflow as described above.(1)

### 4. Construction of the Receptor-Ligand Bipartite Graph

We take as our "Golden Standard" the receptor-ligand dataset from the The Database of Ligand-Receptor Partners (DLRP) [17]. In this dataset, cytokines and interleukins (as well as other ligands) are taken and paired with their corresponding receptor partners. These interactions are then represented in an adjacency matrix where an interaction is represented as a '1' and lack of interaction is represented as a '0'. These are the values we are ultimately trying to predict.

In order to compare the pairings predicted by the combined kernel classifier, we compared the known bipartite receptor-ligand graph (constructed from the known DLRP values) with the predictions from [6] and from the combined kernel classifier. As the combined classifier values are continuous and known values (from DLRP) are binary, it is necessary to determine a threshold value *t *to distinguish between the two classes. The thresholds for each ligand are considered and evaluated as follows. Edges between receptor and ligands are assigned based on the decision function defined in (2), the predicted edge set is then compared to the "*golden standard*" and the precision and recall are calculated. The optimal threshold *t *is then determined by taking the average classifier value of the maximal "*F-measure*" (3) for each ligand. The bipartite graph is then constructed using the edge set resulting from the optimal threshold.(2)(3)

## Results and Discussion

The known tgfβ receptor-ligand set used by Gertz *et al. *[6] consists of 18 known matches. Gertz *et al. *[6] predicted 15 edges, 8 of which were supported and 7 of which were unsupported. In contrast, our tgfβ set consists of 79 known edges, and our approach predicts 90 edges, 60 of which were correct and 30 of which were unsupported. The detailed pairings for this family are shown in Table 1.

**Table 1:** Chemokine receptor-ligand predictions

	*Gertz et al.(2003)*		Iacucci *et al.*	
**Receptor**	**Supported**	**Unsupported**	**Supported**	**Unsupported**

CCR3	SCYA24	--	SCYA26 SCYA7 SCYA5 SCYA15 SCYA11 SCYA13	SCYA3 SCYA17 SCYB6 SCYA4 SCYA21 SCYB5 IL8 SCYA27
CCR1	SCYA2 SCYA8 SCYA13 SCYA7	SCYA11 SCYA1	SCYA3 SCYA7 SCYA8 SCYA5 SCYA15 SCYA13	SCYA26 SCYA17 SCYB6 SCYA4 SCYA21 SCYB5 IL8 SCYA11 SCYA27
CCR5	SCYA3 SCYA4 SCYA5	SCYA14 SCYA15 SCYA23	SCYA3 SCYA4 SCYA5	SCYA26 SCYA17 SCYB6 SCYA21 SCYA15 SCYB5 IL8 SCYA11 SCYA27
IL8RA	SCYB6 SCYB5	GRO1 GRO2 GRO3 PPBP	SCYB6 IL8	SCYA17 SCYA4 SCYA21 SCYA15 SCYA13
CCR4	--	SCYA26	SCYA17	SCYA3 SCYA26 SCYA7 SCYA8 SCYB6 SCYA21 SCYB5 SCYA11 SCYA13 SCYA27
CCR2	--	SCYA21 SCYA19	--	SCYA3 SCYB6 SCYA4 SCYA21 SCYA5 SCYA15 SCYB5 SCYA11
CCR8	SCYA17	SCYA22	--	SCYA3 SCYA26 SCYA7 SCYA8 SCYB6 SCYB5 IL8 SCYA11 SCYA27
CXCR3, GPR9	--	SDF1	SCYA21 SCYA11	SCYA3 SCYA26 SCYA17 SCYA7 SCYA8 SCYB6 SCYA4 SCYA15 SCYB5 IL8 SCYA13 SCYA27
IL8RB	IL8	--	SCYB6 SCYB5 IL8	SCYA3 SCYA17 SCYA7 SCYA8 SCYA4 SCYA21 SCYA5 SCYA15 SCYA11 SCYA13 SCYA27
BLR1, CXCR5	--	MIG SCYB10 SCYB11	--	--
CCBP2, CCR9	SCYA25	--	--	SCYB6 SCYA21
CCR6	--	SCYB13	--	SCYA26
CXCR4	--	SCYA27	--	--
CCR7	--	SCYA20	--	SCYA3 SCYA26 SCYA8 SCYA5 SCYA15 SCYB5

Table of chemokine receptors and their predicted ligand partners, both supported and unsupported partners are shown in both Gertz *et al. *(2003) and the Iacucci *et al. *predictions.

We discover that our predictions for the tgfβ family accurately reconstruct over 76% of the supported edges (0.76 recall and 0.67 precision) of the receptor-ligand bipartite graph defined by the DLRP. In addition, the combined kernel classifier is able to relatively improved upon the Gertz *et al. *[6] work by a factor of approximately two as the Gertz *et al. *[6] work reconstructs 44% of the supported edges (0.44 recall and 0.53 precision) of the receptor-ligand bipartite graph defined by the DLRP. For this family of receptors and ligands, there exists an advantage in our approach to make predictions as we reconstruct more known edges and introduce less noise. Comparing *F*-measures, we see that our method improved upon that of Gertz *et al. *[6] significantly as our method has an *F*-measure of 0.71 while that of Gertz *et al. *[6] has a value of 0.48.

The known chemokine receptor-ligand set used by Gertz *et al. *[6] consists of 63 known matches. Gertz *et al. *[6] predicted 38 edges, 14 of which were supported and 24 of which were unsupported. In contrast, our chemokine set consists of 53 known matches, and our approach predicts 98 edges, 22 of which were correct and 76 of which were unsupported. Our classifier was constructed using ligands which have at least two receptor partners as this greatly improved the precision (0.67 recall and 0.12 precision when all the ligands are used in the classifier). The detailed pairings for this family are shown in Table 2.

**Table 2:** Tgfβ recptor-ligand predictions

	*Gertz et al (2003)*		Iacucci *et al*	
	**Supported**	**Unsupported**	**Supported**	**Unsupported**

TGFBRII	Tgfb2 Tgfb3 Tgfb1	--		
BMPRIa	--	Gdf5	BMP8 BMP4 BMP2 BMP15 BMP3 BMP6 BMP10	Tgfb2 Tgfb3 Tgfb1 INHBA INHA INBB INHC
AMHR	--	--	--	--
BMPRIB	Bmp2 Bmp4	--	BMP8 BMP4 BMP2 BMP15 BMP3 BMP6 BMP10	Tgfb2 Tgfb3 Tgfb1 INHBA INHA INBB INHC
ACTRIIa	ActivinBA	--	BMP7 BMP5 BMP8 BMP4 BMP2 BMP15 BMP3 BMP6 BMP10 INHBA INHA INHBB INHBC	Tgfb3 Tgfb2 Tgfb1
ACTRIIb	ActivinBB	--	BMP7 BMP5 BMP8 BMP4 BMP2 BMP15 BMP3 BMP6 BMP10 INHBA INHA INHBB INHBC	Tgfb2 Tgfb3 Tgfb1
SAX	--	Bmp3	--	--
TKVR	--	Bmp10	--	--
ACTRII	--	DPP	--	--
TGFBRI	--	Bmp7 Bmp6	--	--
BMPRII	--	Gdf8	BMP8 BMP4 BMP2 BMP15 BMP3 BMP6 BMP10	Tgfb2 Tgfb3 Tgfb1 INHBA INHA INBB INHC

Table of tgfβ receptors and their predicted ligand partners, both supported and unsupported partners are shown in both Gertz *et al. *(2003) and the Iacucci *et al. *predictions.

We also find that our predictions for the chemokine family accurately reconstruct over 65% of the supported edges (0.65 recall and 0.23 precision) of the receptor-ligand bipartite graph defined by the DLRP. In addition, the combined kernel classifier is able to relatively improved upon the Gertz *et al. *[6] work by a factor of approximately three as the Gertz *et al. *[6] work reconstructs 22% supported edges (0.22 recall and 0.37 precision) of the receptor-ligand bipartite graph defined by the DLRP. While the precision of the Gertz *et al. *[6] is higher, the recall of our method is about three fold higher. Comparing *F*-measures, we see that our method improved upon that of Gertz *et al. *[6], slightly as our method has an *F*-measure of 0.33 while that of Gertz *et al. *[6], has a value of 0.27.

Qualitatively, the performance of our method also seems to be matching the performance of Gertz *et al. *[6], as the novel interaction of CCR1 with SCY11 [18] reported in their work is also discovered using our method.

**Figure 2:**
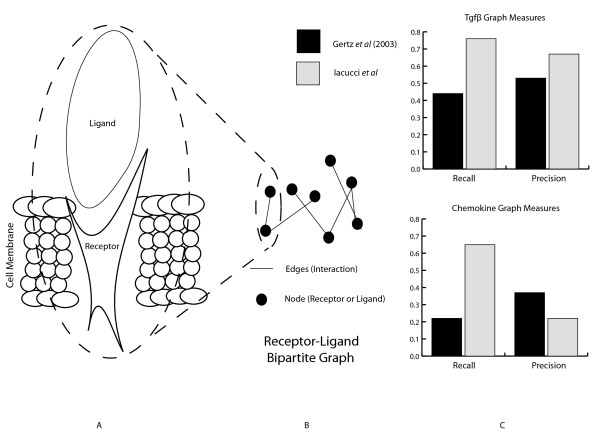
**Schematic of receptor-ligand bipartite graph and performance measures**. (a) Schematic of in-vivo receptor-ligand interaction as found interacting in the cell membrane (b) Bipartite graph schematic of the receptor-ligand interaction network (c) Performance measures of tgfβ and chemokine bipartite graph construction across Gertz *et al. *(2003) and Iacucci *et al. *methods.

The overall results presented here support the notion that kernel learning presents a useful methodology for elucidating receptor-ligand pairing. Using disparate data sources, we propose a combined kernel classifier which is able to reconstruct the majority of known edges in the chemokine and tgfβ receptor-ligand bipartite graphs. In order to evaluate our pairings, we consider the bipartite graph which we construct from our results (see Figure 2). The success of the results are summarized by two performance measures; the recall and the precision of the edges predicted in the tgfβ and chemokine bipartite graphs. The relative performance of each method examined here is evaluated using the *F*-measure.

The combined classifier performs better using the tgfβ family of receptors and ligand than using the chemokine family of receptors and ligands. This can be attributed to two reasons. Firstly, the tgfβ has more positive examples than the chemokine family to train with. Secondly, the tgfβ family is more evolutionarily related while the chemokine family is related by function. Thus, it is more difficult to learn with data from the chemokine family as there is less evolutionarily related structure inherent to the data for the LS-SVM to learn with.

The benefits of the combined kernel classifier method are clear. Foremost in the advantages are the ability to predict multiple ligands for one receptor, which represents an imperative feature for receptor-ligand research. In addition, as the classifier output is continuous, the results can be considered to be prioritized, this presents a major convenience to researchers as often the set of candidate ligands are large and resources to validate few. The major limitation of the method rests in the need to have training examples for receptor-ligands which one is trying to predict. This is particularly true for predicting the pairing in the chemokine dataset as when we consider only ligand candidates with two or more receptor pairings, the precision performance of our method improves (0.79 recall and 0.31 precision) (see Table 1).

The advantage of using the three sub-classifiers instead of a global classifier which combines all features is two fold. The first reason is that the data sources used here are disparate and heterogeneous. A global classifier would require a mapping step which may introduce some noise. The second reason is that using separate sub-classifiers would allow for removal and addition of sub-classifiers. For example, if a better micro-array dataset becomes available in the future, it would be an advantage to be able remove the existing expression-based kernel with one derived from the new dataset without having to the retrain a global classifier. Also, if additional data sources become available, adding an additional sub-classifier based on the new data source would take less time to train than adding the data source and retraining the global classifier.

A practical advantage of using three sub-classifiers in our work became apparent when considering the performance of the individual classifiers versus that of the combined kernel classifier. More specifically, the combined kernel classifier performed equally as well or better than any of the individual classifiers. In the case of the chemokine family, the performance of all three individual classifiers was not nearly as good as the combined kernel classifier. In the case of the tgfβ family, the expression classifier performed nearly as well as the combined kernel classifier (see Additional File 1, Table S1).

## Conclusions

The prediction of receptor-ligand pairings is a difficult and complex task. We have demonstrated that using multiple data sources provide an advantage over single data sources in solving this task. The use of multiple data sources allows us to extend our method as new data becomes available. Among our main contributions we count the ability of our method to prioritize candidate pairs, which represents an imperative feature for receptor-ligand research. As *in-vivo *validation is costly and time consuming, it's important that researchers have a ranking of a, potentially, large number of candidates. In addition, we provide a method which has high recall (0.76 and 0.67) and improved *F*-measures when compared to Gertz *et al. *[6] (0.71 for Iacucci *et al. *vs 0.48 for Gertz *et al. *[6] when evaluationg the tgfβ family and 0.33 for Iacucci *et al. *vs 0.27 for Gertz *et al *[6] when evaluating the chemokine family). Thus, the method is reliable in so far that it will retrieve a large portion of the true positives while not introducing too much noise. As more high throughput data becomes available, we expect to extend the current methodology to accommodate it.

## Competing interests

The authors declare that they have no competing interests.

## Authors' contributions

EI set up the experiments, analyzed the data and wrote the paper. FO participated in designing the study and provided valuable insight and advice. YM and BDM supervised the project. All authors read and approved the final manuscript.

## Supplementary Material

Additional file 1 **Classifier Performance Measures**. The performance of the individual kernel classifiers are displayed in addition to the combined kernel classifier and the Gertz *et al. *(2003) method. Click here for file
