# Supplementary material for: Predicting receptor-ligand pairs through kernel learning
Source: BMC Bioinformatics. 2011 Aug 11;12:336. doi: 10.1186/1471-2105-12-336 (PMC3199765; doi:10.1186/1471-2105-12-336)
Supplement: Additional file 1 — Classifier Performance Measures. The performance of the individual kernel classifiers are displayed in addition to the combined kernel classifier and the Gertz et al. (2003) method. [file 1471-2105-12-336-S1.DOC]

Supplementary Table 1: Classifier Performance Measures:

|  | Measure | Domain | Expression | Phylogenetic | Combined | Gertz |
| --- | --- | --- | --- | --- | --- | --- |
| Chemokine | Recall | 0.34 | 0.39 | 0.36 | 0.64 | 0.22 |
| Family | Precision | 0.17 | 0.11 | 0.06 | 0.22 | 0.37 |
|  | F-Measure | 0.23 | 0.17 | 0.10 | 0.33 | 0.27 |
| Tgfβ | Recall | 0.59 | 0.79 | 0.65 | 0.76 | 0.44 |
| Family | Precision | 0.75 | 0.64 | 0.61 | 0.66 | 0.53 |
|  | F-Measure | 0.66 | 0.70 | 0.63 | 0.71 | 0.48 |

The performance of the individual kernel classifiers are displayed in addition to the combined kernel classifier and the Gertz *et al*. (2003) method.
